# Influence of Fiber Volume Fraction on the Predictability of UD FRP Ply Behavior: A Validated Micromechanical Virtual Testing Approach

**DOI:** 10.3390/ma17194736

**Published:** 2024-09-26

**Authors:** Wael Alhaddad, Minjuan He, Yahia Halabi, Khalil Yahya Mohammed Almajhali

**Affiliations:** 1 Department of Structural Engineering, Tongji University, Shanghai 200092, China; 2 School of Civil Engineering, Southwest Jiaotong University, Chengdu 610031, China

**Keywords:** virtual testing, multiscale modeling, micromechanics, microstructure, RVE size, homogenized properties

## Abstract

Enhancing the understanding of the behavior, optimizing the design, and improving the predictability and reliability of manufactured unidirectional (UD) FRP plies, which serve as primary building blocks for structural FRP laminates and components, are crucial to achieving a safe and cost-effective design. This research investigated the influence of fiber volume fraction ($v_{f}$) on the predictability and reliability of the homogenized elastic properties and damage initiation strengths of two different types of UD FRP plies using validated micromechanical virtual testing for representative volume element (RVE) models. Several sources of uncertainties were included in the RVE models. This study also proposed a modified algorithm for microstructure generation and explored the effect of $v_{f}$ on the optimal sizes of the RVE in terms of fiber number. Virtual tests were systematically conducted using full factorial DOE coupled with Monte Carlo simulation. The modified algorithm demonstrated exceptional performance in terms of convergence speed and jamming limit, significantly reducing the time required to generate microstructures. The developed RVE models accurately predicted failure modes, loci, homogenized elastic properties, and damage initiation strengths with a mean error of less than 5%. Also, it was found that increasing $v_{f}$ led to a concurrent increase in the optimal size of the RVE. While it was found that the $v_{f}$ had a direct influence on homogenized elastic properties and damage initiation strengths, it did not significantly affect the reliability and predictability of these properties, as indicated by low correlation coefficients and fluctuations in the coefficient of variation of normalized properties.

## 1. Introduction

Reliable and predictable behavior of fiber-reinforced polymers (FRPs) is critical to design safe and cost-effective structural FRP components used across various industries, including aerospace, automotive, construction, etc. [1]. The uncertainty in the performance of FRPs is mainly sourced from defects introduced during the manufacturing process, random microstructure morphology, inherent uncertainties in the properties of FRP constituents, and others [2]. By using a robust manufacturing process, the uncertainties related to the behavior of FRPs can be mitigated [3]. Furthermore, comprehending the impact of design parameters such as matrix type, fiber type, and fiber volume fraction ($v_{f}$) on FRP behavior is essential, where by controlling and optimizing these parameters, uncertainties in the mechanical properties of FRPs can potentially be mitigated, leading to an improvement in reliability. For example, the inherent uncertainties in the mechanical properties of natural fibers due to the naturalistic production process of these fibers make the employment of synthetic fibers such as carbon fibers and glass fibers more preferable from the reliability viewpoint because they are produced through controlled process [4,5]. Similarly, the matrix type, whether it falls under the category of thermosets or thermoplastics, could heavily affect the overall FRP reliability.

$v_{f}$ is one of the main design parameters of FRP material. It is evident, both intuitively and from the existing literature [6,7], that increasing the $v_{f}$ would significantly improve the stiffness and strength in the longitudinal direction in a unidirectional (UD) FRP. However, this does not appear to hold true for the transverse direction, particularly concerning strength, because the failure strength in this direction is primarily dictated by the properties of the matrix and fiber–matrix interface. In the light of the inevitable effect of $v_{f}$ on the properties in the transverse direction, pertinent and justifiable questions arise: How and to what extent does the changing of $v_{f}$ affects the various mechanical properties of UD FRP? Would this effect be reflected in the reliability and predictability of UD FRP behavior? Does changing $v_{f}$ produce a consistent effect across different types of UD FRPs?

To answer these questions, a significant number of experiments under different situations and loading scenarios are required. Conducting these experiments is costly and necessitates a significant investment of time and resources. With advancements in technology and increasing computational power, virtual testing through numerical simulation has emerged as a viable alternative to actual testing. This approach offers cost and time savings and enables us to conduct experiments that simulate real-world scenarios and may be difficult to replicate in a laboratory [8,9]. Given the versatility of FRPs, this approach becomes a vital tool for optimizing the design and reliability assessment of the structural FRP components [2]. However, it is crucial to validate the numerical models through actual testing before utilizing them for virtual testing purposes.

**Figure 1:**
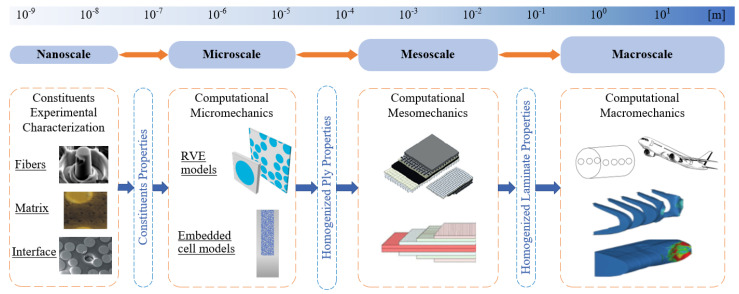
Scheme of bottom-up multiscale strategy for virtual testing of FRP components.

As stated by George Box, “All models are wrong, but some are useful”. In the literature, numerous modeling and virtual testing methods have been developed to aid in the design and optimization of structural FRP components. One of the most effective approaches for developing these virtual testing capabilities is the bottom-up multiscale strategy [10], see Figure 1. This strategy includes three length scale models, viz., the macroscopic, mesoscopic, and microscopic levels models, where different geometric features are presented [11,12]. The bottom-up multiscale strategy was primarily employed in previous research to build numerical models that simulate the actual behavior of FRP laminates and components (i.e., elastic linear behavior, fracture development, and failure mechanism) under different loading scenarios and for different purposes [13,14,15,16,17,18,19,20,21,22]. Also, this strategy was successfully employed in many studies [23,24,25] to develop a framework for multiscale uncertainty quantification and to comprehend how uncertainties at smaller scales, such as th nanoscale, microscale, and mesoscale levels, are reflected in or propagated to the macroscale level. It is worth mentioning other multiscale strategies employed in the literature for similar purposes, such as the $FE^{2}$ Method, Asymptotic Homogenization, Mean Field Approaches, and Transformation Field Analysis, among others. These methods range from purely analytical to highly computational approaches, effectively addressing the complexities of composite behavior, from detailed microstructural characteristics to overall macroscopic responses [26,27].

The fundamental tenet of the framework of the multiscale modeling approaches is the concept of the representative volume element (RVE), which was originally put forward by Hill [28]. RVE models are mainly utilized to predict the homogenized effective properties and damage initiation of FRP plies. A significant challenge associated with the RVE is that it should be geometrically representative of the actual microstructure morphology [29], meaning that the RVE must be statistically equivalent to a real microstructure [30,31]. The effects of the size of an RVE model with random fiber distribution on the predicted homogenized effective properties and localized damage initiation have been investigated over the recent years using different premises related to the source of the RVEs (i.e., algorithmically generated or reconstructed from real micrograph), microstructural features (i.e., fiber shape and micro-voids), constituent materials, finite element model setup, and damage/failure criteria. Most of the studies have drawn varying conclusions regarding the size of the RVE, which is attributed to the inherent differences in their premises. Therefore, it is preferable to conduct a statistical or sensitivity analysis when selecting the optimum RVE size, especially in situations when the premises differ from those adopted in the existing literature.

In the context of the aforementioned background, this study primarily aims to investigate the effect of the $v_{f}$ on the reliability and predictability of FRP ply behavior, viz., orthotropic elastic properties and damage initiation strengths, using a validated micromechanical virtual testing approach. To make this approach more realistic and resemble actual testing, different types of uncertainties and randomness are considered when developing the micromechanical RVE models, such as the inherent uncertainties present in the properties of the FRP constituents’ materials, random fiber arrangement, and uncertainty in fiber diameter.

This study is conducted on two different types of FRPs, AS4/8552 and E-glass/MTM57, to see whether the observed results are the same for various types of FRPs. To achieve the purpose of this research, experimental data for the studied FRP types and constituents are required to define their properties and uncertainties in the numerical models, and to verify these numerical models. Also, it is required to build numerical RVE models by developing a fast algorithm with a high jamming limit to generate a random microstructure. This is crucial for the computational affordability of the adopted virtual testing approach and study the cases of high $v_{f}$. Design of experiment (DOE) coupled with Monte Carlo simulation are needed to create RVE model samples for virtual testing. This study’s significance lies in enhancing the understanding of the behavior, optimizing the design, and improving the predictability and reliability of the manufactured UD FRP plies, which serve as primary building blocks for structural FRP laminates and components, thus achieving a safer and cost-effective design. In addition to the main concern of this study, subsidiary yet important findings can be derived, such as the effect of the $v_{f}$ on the RVE size, because to the authors’ knowledge, there is no available information in the literature about the optimum size of the RVE for different $v_{f}$ values, or whether the $v_{f}$ has an impact on the RVE size in the first place.

In the rest of this article, the most common sources of uncertainty that affect the performance of FRPs and their modeling methods are outlined to provide insights for enhancing virtual testing realism. Moreover, the considered types of FRP materials, the experimental micromechanical characterization of their constituents’ properties, and the development of RVE models and full factorial DOE incorporated with Monte Carlo simulation are detailed. Lastly, the results related to model validity checking and to the effect of $v_{f}$ on RVE size and on the reliability and predictability of homogenized properties and damage initiation strengths are discussed and interpreted.

## 2. Sources of Uncertainty in Structural FRP and Their Modeling Methodologies

The presence of uncertainties at different scales in manufacturing structural FRP components (i.e., the constituent level, ply level, laminate level, and component level) has raised significant concerns regarding the safety of FRP structures. The uncertainty in the performance (i.e., mechanical and thermal properties) of structural FRPs at the ply level (microscale level) results from different uncertainty sources. In this study, the first considered source of uncertainty is the inherent uncertainties in the mechanical properties of FRP constituents (fibers and matrix). The experimental works in the literature [32,33,34,35,36] have concluded that the probability density function (PDF) of Weibull distribution is the fittest PDF to model the uncertainties in the mechanical properties of the fibers, specifically fibers’ tensile strength, which is explained by the weakest link theory [37]. Meanwhile, for the polymer matrix stiffness and strength, the PDF of Gaussian distribution is good enough [38,39], especially if the matrix material is isotropic. The second considered source is the variations in the fiber diameter. These variations slightly alter the fiber content in the FRP and the size of the interface between the fibers and matrix. In the existing literature, the uncertainty of fiber diameter was identified and measured under the electronic microscope, and modeled using the PDF of the lognormal distribution [40].

The third considered source is fiber spatial distribution (microstructure morphology). The random spatial arrangement of fibers influences the flow of the polymer matrix. This can result in high accumulation of the matrix in certain areas and low accumulation in others, potentially leading to the formation of voids [41]. This dispersion in local fiber content causes inhomogeneity in stress and strain distribution, ultimately leading to additional uncertainties and randomness in the mechanical properties of the FRP material. Several algorithms for numerically generating a random microstructure were proposed in the literature to consider the uncertainty associated with fiber spatial distribution. For example, the Random Sequential Adsorption (RSA) method [42,43,44,45,46,47], growth-based algorithms [48,49] molecular dynamics-based algorithms [50,51], an algorithm based on a constrained optimization formulation [52], etc. Each of these algorithms has its limitations in terms of jamming limit and execution time. Therefore, several modified versions of these algorithms were proposed by the researchers to overcome these limitations to some extent. In line with this context, this research also contributes by presenting a modified version of an algorithm based on a constrained optimization formulation, as adopted in the study by Pathan et al. [52]. The modifications to the algorithm mainly aim to speed up the execution time, i.e., reduce the computational cost, without lowering the jamming limit obtained by the original version of this algorithm, because the computational cost is critical for the visibility and practicality of the application of virtual testing and reliability analysis, where hundreds or thousands of numerical models need to be generated and analyzed. The concept, equations, and pseudocode of the algorithm are presented in Section 4.1.

The uncertainty sourced from the variations in the actual $v_{f}$ is implicitly accounted for by considering the three prementioned sources of uncertainties. Other sources of uncertainties that stem from defects that occur during the manufacturing process of FRP composites and products, such as voids in the matrix, fiber misalignment, and fiber waviness, are not considered in this study.

## 3. Materials and Micromechanical Characterization of the FRPs’ Constituents

The design of two main types of FRPs are investigated in this study: carbon fiber-reinforced polymer and glass fiber-reinforced polymer. The considered constituents of these types of FRPs are AS4 carbon fibers, E-glass fibers, 8552 epoxy, and MTM57 epoxy. The required micromechanical and macromechanical experimental techniques to characterize each of the constituents are as follows.

### 3.1. Microstructure Analysis and Image Processing

**Figure 2:**
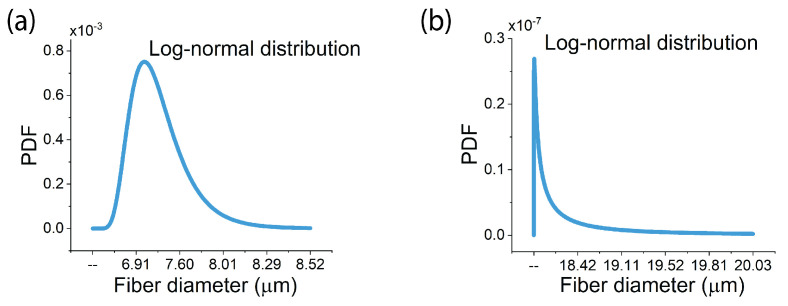
(**a**) The distribution of fiber diameter of AS4/epoxy; (**b**) the distribution of fiber diameter of E-glass/epoxy.

To analyze the microstructure of the studied types of FRPs, a visual inspection using an optical microscope or scanning electronic microscope (SEM) is required to obtain the necessary micrographs. Fortunately, plenty of micrographs of the AS4/epoxy and E-glass/epoxy are available in the literature [13,18]. Therefore, in this research, by conducting image processing on these micrographs using *open-source Java-based ImageJ (1.54d)* software, the microstructure of the AS4/epoxy and E-glass/epoxy was analyzed and the diameters of the fibers were measured and fitted to the PDF of the lognormal distribution to model the uncertainty in fiber diameter. Figure 2a,b display the lognormal distribution of fiber diameter for AS4 and E-glass fibers, respectively. The mean and standard deviation of these distributions are 7.28±0.45μm for the AS4 fibers and 17.76±1.96μm for E-glass fibers.

### 3.2. Characterization of Fibers

**Figure 3:**
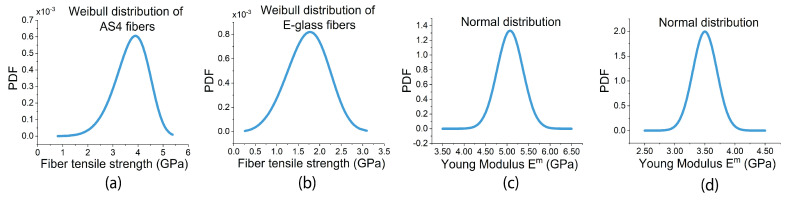
Weibull models for the uncertainties in the tensile strength of (**a**) AS4 and (**b**) E-glass fibers; normal distribution models for the uncertainties in the Young modulus of (**c**) 8552 and (**d**) MTM57 epoxies.

Due to their amorphous structure, E-glass fibers can be considered as elastic isotropic solids [53]. Meanwhile, AS4 fibers behave as elastic transversely isotropic solids, where the isotropy plane is defined by directions 2–3 and direction 1 corresponds to the fiber direction [54]. The longitudinal properties are typically obtained from direct loading and the testing of tows and/or filaments of these fibers. Example tests include the single-fiber test [53], microcomposite testing [55], the in situ micropillar compression test [56], etc. Meanwhile, the transverse properties of the fibers can be obtained from the single-filament transversal compression test [57,58] or most commonly using analytical models based on homogenization theories or computational micromechanical models [15]. However, the mechanical properties of AS4 and E-glass fibers have been extensively investigated and documented in the literature [19,56,59], see Table 1, and the available information was sufficient to model the uncertainties of the fibers’ strength using the PDF of Weibull distribution, as shown in Figure 3a,b.

### 3.3. Characterization of Matrix

**Figure 4:**
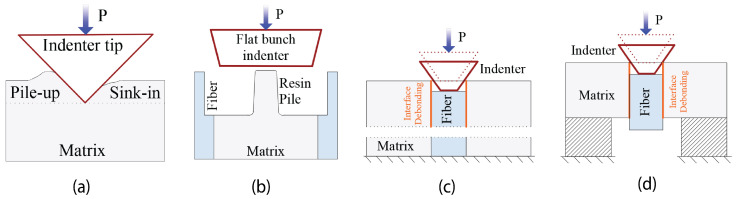
Schemes of (**a**) instrumented nanoindentation test; (**b**) micropillar compression test; (**c**) push-in test; and (**d**) push-out test.

The tensile strength and elastic properties of epoxy matrices can be approximately determined via macroscale ex situ tests on the neat epoxy coupons. Meanwhile, employing in situ micromechanical tests, such as instrumented nanoindentation [60] (see Figure 4a) and micropillar compression techniques [61] (see Figure 4b), would be preferred to obtain more representative mechanical properties for the matrix, fibers, and interface within the FRP composite material as manufactured. However, the mechanical properties of 8552 and MTM57 epoxies matrices have also been extensively investigated and reported in the literature by researchers and material suppliers [13,14,18,20,56]. These properties are listed and summarized in Table 2. Based on this, the uncertainties of the Young modulus ($E^{m}$) of these matrices were modeled using the PDF of the normal distribution, as shown in Figure 3c,d.

### 3.4. Characterization of Fiber–Matrix Interface

The properties of the fiber–matrix interface in FRP composite can be examined and characterized through either ad hoc micromechanical tests or in situ micromechanical tests. The most common ad hoc micromechanical tests are the single-fiber fragmentation test [62], fiber pull-out test [63], microbond test [64], and single compression test. The in situ micromechanical tests, such as push-in and push-out tests [65], see Figure 4c,d, have a primary advantage over the ad hoc ones, because they are conducted on the actual composite, which means that the actual constraining effect of the surrounding fibers and the residual stress in the polymer matrix are taken into consideration. Regardless of the type of test, both ad hoc and in situ micromechanical tests are mainly meant to obtain the interface shear strength ($S^{c}$), while other properties such as interface normal strength ($N^{c}$), Fracture energy mode I ($G_{n}^{c}$), and Fracture energy mode II ($G_{s}^{c}$) cannot be obtained using these tests, but usually they are estimated or calibrated through numerical simulation. As an approximation, the interface normal strength can be calculated as 2/3 of the interface shear strength [66]. In this research, the mechanical properties of the fiber–matrix interface of AS4/epoxy and E-glass/epoxy composites were obtained from literature sources [13,14,20,56]; however, the missing properties were estimated and calibrated using numerical models, as seen in Section 4. Table 3 presents these properties for AS4/epoxy and E-glass/epoxy.

## 4. Developing the Computational Microscale Models

The 3D computational microscale models for virtual testing were developed using the periodic RVE method containing the randomness and uncertainties of the fiber distribution, fiber diameter, $v_{f}$, and constituents’ properties. Periodic RVE models are suitable for use only during the elastic regime to analyze uniform stress and strain fields, compute homogenized elastic properties of UD FRP plies, and predict the damage initiation strengths for these plies under various loading conditions. This makes periodic RVE models sufficient for the scope of this research. The development of the periodic RVE models is clarified in the following subsections.

### 4.1. Microstructure Generation

**Figure 5:**
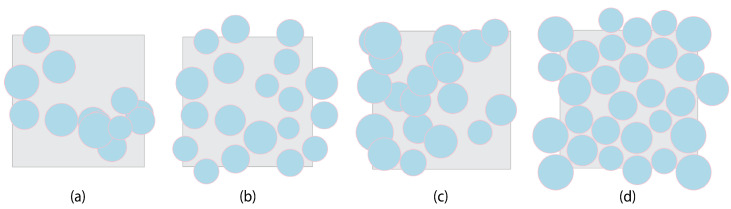
Spatial distribution of the fibers; (**a**) initial guess for $v_{f}=50\%$; (**b**) final arrangement for $v_{f}=50\%$; (**c**) initial guess for $v_{f}=75\%$; (**d**) final arrangement for $v_{f}=75\%$.

This research proposes an enhanced version of an algorithm based on a constrained optimization formulation, specifically minimization of the fiber overlapping function, as adopted in the study by Pathan et al. [52]. The algorithm consists of two main stages:

***Initial Guess stage:*** In this stage, the algorithm starts by randomly generating $N_{f}$ diameters ($D_{i}$) of fibers from the corresponding lognormal distribution of fiber diameter (e.g., Figure 2a,b), where $N_{f}$ is the targeted number of fibers in the RVE model. Then, the size of the RVE (L×H) is calculated according to the total area of fibers and the targeted $v_{f}$, see Equation (1). Then, according to uniform distribution, the algorithm proceeds to randomly generate, at the same time, the coordinates ($x_{i}$, $y_{i}$) for the centers of all the $N_{f}$ fibers within the range of the RVE dimension (see Equation (2)). After this, for every fiber that intersects the boundaries of the RVE, a twin fiber is created and located such to guarantee the periodicity of the microstructure. Then, the diameter of each fiber is enlarged with the value of 1μm. Figure 5a,c shows examples of initial arrangements of randomly placed fibers; the fibers overlapping are clear.

(1) $$L\times H=\pi \sum_{i=1}^{N_{f}}\frac{D_{i}^{2}}{4v_{f}}$$

(2) $$0\leq x_{i}\leq L\;\mathrm{and}\;0\leq y_{i}\leq H\;\forall i\in N_{f}$$

***Optimization stage:*** To prevent fibers overlapping, the algorithm in this stage will formulate the optimization problem according to Equations (3)–(6). These equations represent a feasibility problem; Equation (3) represents the boundaries of the design space; Equation (4) is a nonlinear inequality constraint for representing the condition that fibers should not overlap and keep minimum tolerance distance between the fibers (*l_f min_*); while Equation (5) is also an inequality constraint that guarantees a minimum tolerance distance (*l_e min_*) between the fibers and the edges of the RVE.

(3) $$-0.25D_{i}\leq x_{i}\leq L+0.25D_{i}\;\mathrm{and}\;-0.25D_{i}\leq y_{i}\leq H+0.25D_{i}\;\forall i\in N_{f}$$

(4) $$\sqrt{{\left(x_{i}-x_{j}\right)}^{2}+{\left(y_{i}-y_{j}\right)}^{2}}\geq 0.5\left(D_{i}-D_{j}\right)+l_{f_{\min}}\;\forall i,j\in N_{f}\;\;i\neq j$$

(5) $$\left.\begin{array}{cc} \mathrm{Left}\;\mathrm{edge}: & |x_{i}-0.5D_{i}|\geq l_{e_{\min}}\times D_{i}\\ \mathrm{Right}\;\mathrm{edge}: & |x_{i}-0.5D_{i}-L|\geq l_{e_{\min}}\times D_{i}\\ \mathrm{Top}\;\mathrm{edge}: & |y_{i}-0.5D_{i}-H|\geq l_{e_{\min}}\times D_{i}\\ \mathrm{Bottom}\;\mathrm{edge}: & |y_{i}-0.5D_{i}|\geq l_{e_{\min}}\times D_{i} \end{array}\right\}$$

(6) $$F=\sum_{k=1}^{N_{c}}\alpha_{k}^{2}$$

A feasible solution that could satisfy these constraints can be obtained by minimizing the penalty function (Equation (6)), which assigns a non-negative penalty ($\alpha_{k}$) for each constraint violation at each fiber, i.e., considering a generic form of the constraints on the coordinates of the k−th fiber $g_{k}\left(x\right)\geq b_{k}$, the penalty associated with the violation of this constraint is $\alpha_{k}={\langle b_{k}-g_{k}\left(x\right)\rangle }^{2}$. Then, the formulated feasibility problem is solved and the feasible solution is obtained by minimizing the penalty function using the **L-BFGS-B** algorithm [67] that is based on the quasi-Newton optimization method. The algorithm will be terminated when the value of the penalty function is equal to zero or the maximum number of iterations is reached. To validate the feasibility of the obtained optimum solution, the original diameter of each fiber is retrieved by subtracting the previously added value of 1μm, and the value of the penalty function is recalculated. If it is equal to zero, then the solution is feasible; otherwise, the solution will be discarded, and the algorithm will go back to the initial guess stage and start searching for a new solution. Figure 5b,d illustrate examples of the final arrangements of randomly placed fibers in the RVE. The pseudocode of this algorithm is shown in Algorithm 1, which is scripted and executed in Python.

When the targeted $v_{f}$ is high (more than 60%), the number of feasible solutions decreases, making it difficult to achieve a penalty function of zero value. This is where the added value to the diameter of each fiber plays a role. It helps in speeding up the process of discarding the infeasible solutions and ensuring that the penalty function becomes zero when removing this value from the good solutions with low penalty function values. Therefore, the algorithm exhibited exceptional performance in terms of convergence speed, albeit with a slight trade-off in the jamming limit, which reached approximately 77%. This value is slightly lower than the 80% jamming limit in the reference [52]. However, the convergence speed is significantly higher, as it took less than 5 minutes on average to generate a feasible microstructure of size 50×50 with a $v_{f}$ of 65%, which is a lower time in comparison with algorithm of reference [52], which took 18 min on average to generate a similar microstructure with the same size and $v_{f}$. This is critical and highly beneficial for uncertainty analysis using virtual testing, particularly when a large number of microstructures need to be generated.
**Algorithm 1** Pseudocode for generating microstructure1:Defining the input parameters: $N_{f}$, $v_{f}$, PDF of *D*, $l_{f_{\min}}$, $l_{e_{\min}}$2:**While** Final_penalty > 0 and rounds < 10^3^:3:   *D* = Generating diameter of $N_{f}$ fibers4:   Calculate *L* & *H* (Equation (1))5:   Generating $N_{f}$ random coordinates $\left(x_{i},y_{i}\right)$ (Equation (2))6:   Generating twin fibers for each fiber intersects the boundaries of RVE7:   *D* = D+1μm8:   **For** *i* in *D*:9:     Assign the *i*-th fiber coordinate as design variable10:     Defining boundaries (Equation (3))11:     Defining constraints (Equations (4) and (5))12:     Defining and calculating the penalty function (Equation (6))13:     **if** penalty > 0:14:        Solving the optimization problem (***minimize*** function & ***L-BFGS-B method***)15:        Obtaining optimum $\left(x_{i},\;y_{i}\right)$16:   D=D−1μm17:   Final_penalty = Recalculate the penalty function (Equation (6))18:   rounds += 119:   **if** Final_penalty = 0:20:     export the fibers coordinates and diameters to text file21:     plot the RVE with the randomly distributed fibers

### 4.2. Periodic Boundary Conditions (PBCs) and Displacement Boundary Conditions

**Figure 6:**
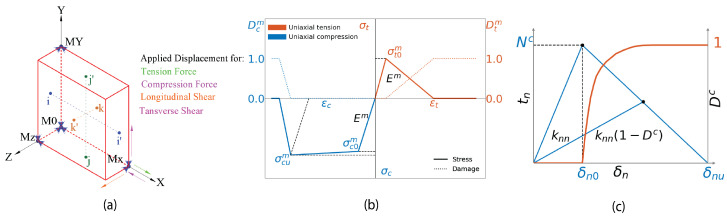
(Color online) (**a**) Diagram clarifies the derivation of PBC equations; (**b**) a constitutive response diagram of the epoxy matrix [69]; (**c**) a diagram of the traction–separation law of the fiber–matrix interface (normal response and damage variable evolution) [69].

The periodic boundary conditions (PBCs) in a general 3D RVE can be expressed in terms of the displacement constraints in vector form, where the relative displacement between the matched nodes on the opposite faces of the RVE (${\vec{U}}_{x}$, ${\vec{U}}_{y}$, and ${\vec{U}}_{z}$) should be equal to the relative displacement between a set of master nodes (typically $M_{0},M_{x},M_{y},M_{z}$) laying on the opposite faces. According to Figure 6a, the PBCs can be expressed mathematically as shown in Equations (7)–(9), where *L*, *H*, and *t* are the dimensions of the RVE in the *x*, *y*, and *z* directions, respectively.

(7) $$\vec{u}\left(L,y,z\right)-\vec{u}\left(0,y,z\right)={\vec{u}}_{M_{X}}-{\vec{u}}_{M_{0}}={\vec{U}}_{x}$$

(8) $$\vec{u}\left(x,H,z\right)-\vec{u}\left(x,0,z\right)={\vec{u}}_{M_{Y}}-{\vec{u}}_{M_{0}}={\vec{U}}_{y}$$

(9) $$\vec{u}\left(x,y,t\right)-\vec{u}\left(x,y,0\right)={\vec{u}}_{M_{Z}}-{\vec{u}}_{M_{0}}={\vec{U}}_{z}$$

PBCs were imposed in Abaqus through modifying the keyword of the model to include the equation of PBCs from a text file. While PBCs are necessary to maintain the periodic nature of the RVE, displacement boundary conditions are imposed to generate stresses. Thus, an appropriate set of displacement boundary conditions was applied on the master nodes ($M_{0},M_{x},M_{y},M_{z}$) for different loading cases.

### 4.3. Constitutive Models and Discretization of Fiber, Matrix, and Interface

AS4 fibers were modeled as linear, elastic, and transversally isotropic solids. Five independent elastic constants ($E_{1}^{f}$, $E_{2}^{f}$, $G_{12}^{f}$, $G_{23}^{f}$, $\nu_{12}^{f}$) and two thermal expansion coefficients ($\alpha_{1}^{f}$, $\alpha_{2}^{f}$) were defined as clarified in Table 1 to take the anisotropic behavior between the longitudinal and transverse directions of the AS4 fibers into consideration. Meanwhile, E-glass fibers were modeled as linear isotropic elastic solids with the elastic properties shown in Table 1. Both AS4 and E-glass fibers were discretized and meshed using 6-node fully integrated wedge elements (C3D6).

The epoxy matrix behaves as a frictional material, exhibiting brittle behavior under tensile loads while exhibiting pronounced plastic behavior in compression. As the yield surface is influenced by hydrostatic pressure, it can be modeled using a Drucker–Prager yield criterion, assuming isotropic elastic–plastic behavior [68]. The main features of the Drucker–Prager constitutive model are the pressure-dependent yield surface evolution and the distinction between compressive and tensile damage evolution. Figure 6b shows a scheme of the damage–plasticity model [69], which is a modification of the Drucker–Prager plasticity yield surface model [68]. This model necessitates defining the mechanical response under uniaxial tension and compression in addition to the evolution of the yield surface (plasticity) and material degradation (damage). However, the values of the required parameters ($E^{m}$, $\nu^{m}$, $\alpha^{m}$, $\sigma_{t}^{m}$, $\psi^{m}$, and $\sigma_{yc}^{m}$) to define the elastic and plastic behavior for both 8552 and MTM57 epoxy matrices using the Drucker–Prager constitutive model were taken as specified in Table 2. The matrix was discretized and meshed using eight-node reduced integration linear bricks with hourglass control (C3D8R).

Fiber–matrix interface behavior and failure mechanisms (debonding) were modeled using a classical cohesive zone method [70,71,72]. Cohesive elements were inserted at the fiber–matrix interface. The behavior of these elements was defined by the mixed-mode traction–separation law, and their damage was governed by the quadratic stress criterion (Equation (10)) [13,66].

(10) $${\left(\frac{\langle t_{n}\rangle }{N^{c}}\right)}^{2}+{\left(\frac{t_{s}}{S^{c}}\right)}^{2}+{\left(\frac{t_{t}}{S^{c}}\right)}^{2}=1$$

where $t_{n}$, $t_{s}$, and $t_{t}$ are the normal and shear components of the traction vector; 〈·〉 denotes the Macaulay brackets, emphasizing that a purely compressive stress state does not initiate damage [73]. As seen in Figure 6c, initially, the cohesive element behavior was linear elastic with very high penalty stiffnesses ($k_{n}^{c}$, $k_{s}^{c}$). When the damage was initiated, a linear softening was induced to capture the degradation of the stiffness up to complete failure. The energy dissipation was computed through the power law under mixed-mode loading [69]. However, the values of the required parameters to define the described constitutive model of fiber–matrix interface behavior were taken as specified in Table 3. It is also worth mentioning that the fiber–matrix interface was meshed using eight-node three-dimensional cohesive elements (COH3D8).

### 4.4. Loading Cases and Steps

To derive the homogenized elastic properties, thermal expansion coefficients, and damage initiation strengths of the investigated FRP plies, various loading scenarios were applied to the periodic RVE model. Each loading case was designed to capture specific properties.

For determining the homogenized elastic properties—namely, Young’s moduli, Poisson’s ratios, and shear moduli—alongside the homogenized thermal expansion coefficients in the three directions, a loading case of six mechanical steps and one thermal step was applied to the periodic RVE. In each of the six mechanical steps, unit stress was applied to distinct RVE faces, oriented in different directions (x,y,z) under suitable boundary conditions, to obtain the homogenized elastic properties. In the thermal step, a homogeneous uniform temperature field with a magnitude of 1 °C was defined, enabling the determination of homogenized thermal expansion coefficients. It should be noted that, in this loading case, the RVE and its constituents are assumed to behave as a linear elastic material, because the inelastic behavior does not play a role in determining the homogenized elastic properties of the FRP plies.

To obtain the damage initiation strengths under uniaxial transverse tension and compression, two distinct loading cases were devised: one applying a 10% strain and the other a −10% strain in the transverse direction. Similarly, two additional loading cases were devised by applying 0.1 strain in the transverse and longitudinal directions to obtain the damage initiation strengths under transverse and longitudinal shear. However, a homogeneous uniform thermal step was applied initially to consider the effect of the residual thermal stresses stemming from the mismatch of the thermo-elastic constants of fibers and the matrix on the damage initiation strength of the FRP plies. This thermal step modeled the cooling process from the curing temperature (120 °C) to the service temperature (20 °C).

### 4.5. RVE Size Validation

The size of the RVE should guarantee that it geometrically represents the actual microstructure, meaning the RVE must be statistically equivalent to the real microstructure. A too-small RVE will not be geometrically representative, while a too-large RVE will be computationally expensive. Achieving a good geometric representation while keeping computational costs reasonable requires optimizing the size of the RVE. The optimum size for the RVE is the smallest size that encompasses all the irregularities affecting stress distribution. This ensures that the obtained homogenized properties of the FRP are independent of the RVE size and accurately represent the macroscopic constitutive response. However, there are no specific procedures to predict the optimum size of the RVE for analyzing a particular FRP. It is usually confirmed by a sensitivity study for the convergence of the following: (i) RVE Micromechanical Features, such as homogenized properties, damage initiation strengths, strain energy, energy density, etc., or (ii) RVE Statistical Microstructure Descriptors, classified into Geometric Descriptors and Statistical Functions.

In this research, the optimum size of the RVE was obtained by evaluating the convergence of the effective properties and damage initiation strength. The RVE size was measured and represented in terms of fiber number ($N_{f}$) for a specific volume fraction ($v_{f}$) and a consistent aspect ratio of one (H/L=1), i.e., square-shaped RVEs. Additionally, it is important to note that in all cases, the thickness of the RVE was set to a constant value of t=1μm.

### 4.6. Solver, Postprocessing, and Implementation

The Abaqus standard solver [69] was employed to analyze the 3D periodic RVE model. The mesh size was defined using a sensitivity analysis. The obtained analysis results were subjected to postprocessing based on the mechanics of materials equations to calculate the homogenized elastic properties and damage initiation strengths.The development and analysis of the 3D periodic RVE model, incorporating the previously described random microstructure, periodic boundary conditions (PBsC), constitutive models, loading cases, sizes, and postprocessing, were scripted and implemented in Python with the help of Abaqus modules, such as ***abaqus***, ***abaqusConstants***, and ***caeModules***. Utilizing scripting to automate the development and analysis of RVE models offers significant flexibility and ensures an efficient workflow for iterative analysis and experimentation.

## 5. Full Factorial DOE and Monte Carlo Simulation for Virtual Tests

**Figure 7:**
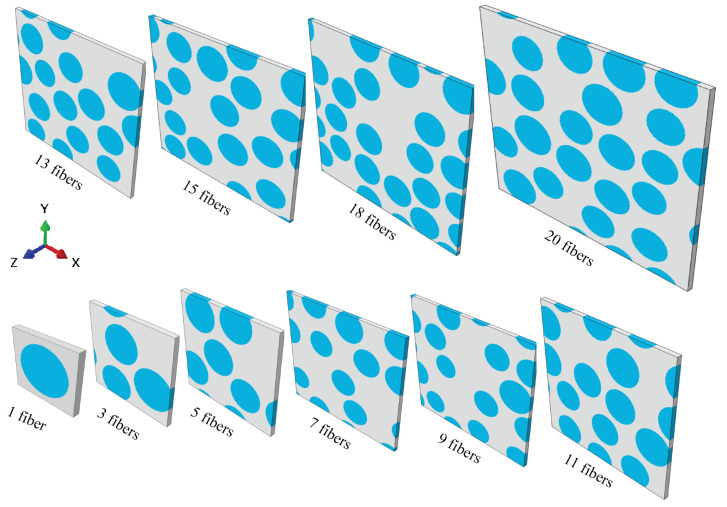
RVE model specimens of different sizes for AS4/epoxy (similar RVE can be applied to E-glass/epoxy considering E-glass geometry).

To systematically validate the size of the developed RVE models and assess the effect of $v_{f}$ on the predictability and reliability of FRP behavior, while accounting for various sources of randomness and uncertainties, a full factorial design of experiments (DOE) of 2 factors, 10 levels, and 10 replicates, coupled with Monte Carlo simulation, was conducted for designing the virtual tests. The two factors under consideration were $v_{f}$ and RVE size (number of fibers, $N_{f}$), each with 10 levels as detailed in Table 4. This full factorial DOE was applied for virtually testing two types of FRPs (AS4/8552 and E-glass/MTM57) to determine if the findings hold true for different types of FRPs. The assessed responses include the homogenized elastic properties and damage initiation strengths. Monte Carlo simulation was employed to generate RVE samples based on the probability density functions (PDFs) of the considered types of uncertainties (Figure 2 and Figure 3). Consequently, considering the studied types of FRPs, i.e., AS4/8552 and E-glass/MTM57, two samples of RVE models were generated, each sample containing 1000 specimens of RVE models. In total, 2000 RVE models were analyzed. Figure 7 shows some RVE model specimens of different sizes.

## 6. RVE Model Validation

The developed RVE models should be validated against actual experimental data (i) to determine the optimum size of the RVE ($N_{f}$) that gives converged predictions of different mechanical properties and (ii) to check the accuracy of these predictions. However, actual experimental data are not available for all studied cases of different $v_{f}$. Therefore, by establishing the validity of certain models for which experimental data are accessible, the validity of the other models can be inferred. In the literature, effective mechanical properties have been obtained from actual tests conducted on macroscopic coupons extracted from (i) UD AS4/8552 laminate with a $v_{f}$ of 58% [74,75,76] and (ii) UD E-glass/MTM57 laminate with a $v_{f}$ of 54% [20,59,77,78,79]. These effective properties are presented in Table 5. The validity of the models was evaluated and discussed from the following perspective.

### 6.1. Efficiency and Execution Time of the Algorithm for RVE Model Generation and Analysis

The scripted codes to generate and analyze the RVE models were executed on a desktop computer equipped with an *AMD Ryzen* 5 600*X* 6-*CoreProcessor* (AMD, Santa Clara, CA, USA) running at 3.70 GHz with 16 GB of RAM. The analysis results revealed that the scripted codes efficiently generated and analyzed the RVE models under the predefined loading cases within reasonable timeframes. The processing times ranged from less than 7 min for the smallest RVE to 45 min for the largest RVE of the AS4/8552 composite, and from less than 10 min for the smallest RVE to 102 min for the largest RVE of the E-glass/MTM57 composite. Furthermore, the analysis speed varied across loading cases. The first loading case exhibited the fastest performance, taking only a few seconds regardless of the RVE size due to the elastic linear nature of the analysis in this loading case. However, the analysis pace slowed down for subsequent loading cases (transverse tension and compression and longitudinal and transverse shears) due to the inelastic nonlinear nature of the analysis in these loading cases.

### 6.2. Optimum RVE Size and Model Prediction Validity from the Perspective of Homogenized Elastic Properties and Damage Initiation Strengths

**Figure 8:**
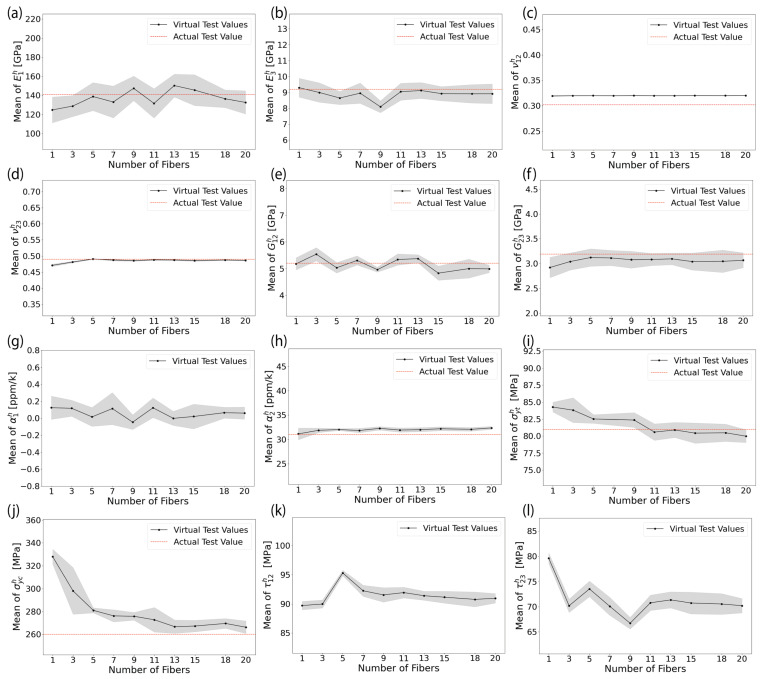
(Color online) The homogenized elastic properties and failure initiation strengths obtained from analyzing different sizes of RVE models of AS4/8552: (**a**) $E_{1}^{h}$; (**b**) $E_{2}^{h}$; (**c**) $\nu_{12}^{h}$; (**d**) $\nu_{23}^{h}$; (**e**) $G_{12}^{h}$; (**f**) $G_{23}^{h}$; (**g**) $\alpha_{1}^{h}$; (**h**) $\alpha_{2}^{h}$; (**i**) $\sigma_{yt}^{h}$; (**j**) $\sigma_{yc}^{h}$; (**k**) $\tau_{12}^{h}$; (**l**) $\tau_{23}^{h}$ (see Table 5 for actual test values [20,59,74,75,76,77,78,79]).

**Figure 9:**
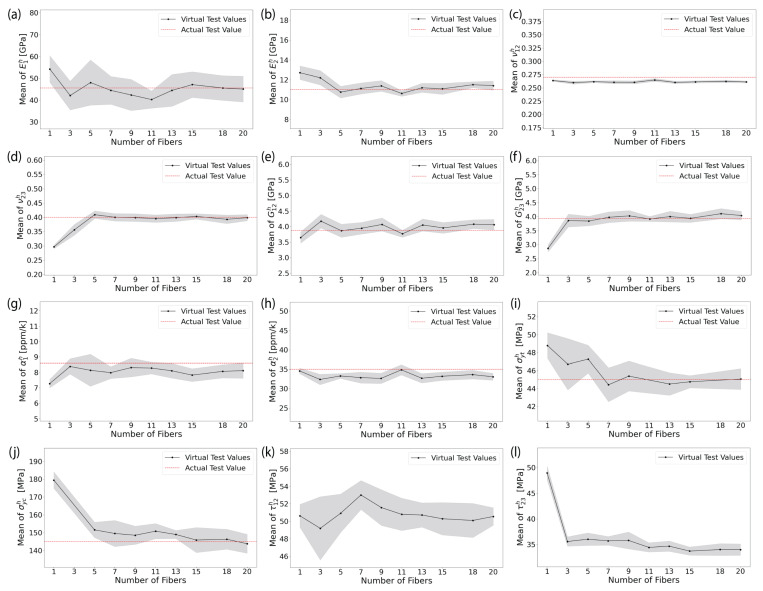
(Color online) The homogenized elastic properties and failure initiation strengths obtained from analyzing different sizes RVE models of E-glass/MTM57: (**a**) $E_{1}^{h}$; (**b**) $E_{2}^{h}$; (**c**) $\nu_{12}^{h}$; (**d**) $\nu_{23}^{h}$; (**e**) $G_{12}^{h}$; (**f**) $G_{23}^{h}$; (**g**) $\alpha_{1}^{h}$; (**h**) $\alpha_{2}^{h}$; (**i**) $\sigma_{yt}^{h}$; (**j**) $\sigma_{yc}^{h}$; (**k**) $\tau_{12}^{h}$; (**l**) $\tau_{23}^{h}$ (see Table 5 for actual test values [20,59,74,75,76,77,78,79]).

The trend in the estimated mean homogenized elastic properties and damage initiation strengths of AS4/8552 and E-glass/MTM57 UD plies using computational homogenization of RVEs of different sizes (i.e., different $N_{f}$) are illustrated in Figure 8 and Figure 9, respectively. The depicted homogenized properties in these figures represent the average of these properties from ten trials for each RVE size, accompanied by an error band that signifies a 95% confidence interval of the average. Additionally, the values of the homogenized elastic properties obtained from actual experiments are also depicted in the same figures for comparison and validation.

The results of the longitudinal homogenized elastic moduli $E_{1}^{h}$, as shown in Figure 8a and Figure 9a, exhibit fluctuations and do not display convergence with an increasing number of fibers. The observed fluctuations in values and the wide confidence interval can be attributed to the randomness considered in the properties of the constituents, i.e., fiber properties and polymer matrix properties. A similar observation and conclusion can be extended to the longitudinal homogenized shear moduli, i.e., out-of-plane $G_{12}^{h}$, as shown in Figure 8e and Figure 9e. However, for the homogenized Poisson’s ratios in the longitudinal direction, i.e., out-of-plane $\nu_{12}^{h}$, the fluctuations arising from the considered randomness in the constituent properties are less evident, as illustrated in Figure 8c and Figure 9c. This is because the Poisson’s ratio in transversally isotropic material is calculated according to the relationship $\nu =\frac{E}{2G}-1$, and the ratio of the fluctuated values of Young’s and shear moduli remains relatively constant. The predicted transverse homogenized elastic moduli $E_{2}^{h}$, as shown in Figure 8b and Figure 9b, tend to converge for RVEs with more than 11 fibers. Meanwhile, the in-plane or transverse homogenized shear moduli $G_{23}^{h}$ and Poisson’s ratios $\nu_{23}^{h}$ tend to converge for RVEs with more than 5 fibers, as evident in Figure 8d,f and Figure 9d,f. Regarding the homogenized coefficients of thermal expansion in the longitudinal and transverse directions $\alpha_{1}^{h}$ and $\alpha_{2}^{h}$, as clarified in Figure 8g,h and Figure 9g,h, they seem largely unaffected by variations in the size of the RVE. The observed fluctuations in their values can also be attributed to the randomness in the constituent properties. Overall, the obtained homogenized elastic properties of the FRP plies were transversally isotropic in the planes perpendicular to the fiber, i.e., $E_{2}^{h}\approx E_{3}^{h}$, $G_{12}^{h}\approx G_{13}^{h}$, $\nu_{12}^{h}\approx \nu_{13}^{h}$, and $\alpha_{2}^{h}\approx \alpha_{3}^{h}$. It is important to note that not all results are presented in this paper due to space constraints.

From the perspective of damage initiation strengths, the homogenized transverse tensile and compressive strengths $\sigma_{yt}^{h}$ and $\sigma_{yc}^{h}$, as illustrated in Figure 8i,j and Figure 9i,j, exhibit a descending trend with an increasing number of fibers. This trend can be attributed to the fact that as the number of randomly distributed fibers increases, more stress concentration regions are formed, consequently leading to a reduction in failure stress. This observation further validates the accuracy and robustness of the developed models. The $\sigma_{yt}^{h}$ and $\sigma_{yc}^{h}$ tend to converge for the RVEs with more than 11 fibers. Similarly, the homogenized longitudinal and transverse shear strengths $\tau_{12}^{h}$ and $\tau_{23}^{h}$ appear to converge for the RVEs with more than 11 fibers, as shown in Figure 8k,l and Figure 9k,l.

From the perspective of prediction accuracy in comparison with actual experimental data, the maximum mean error after convergence for all scenarios in predicting the homogenized elastic properties and damage initiation strengths did not exceed 5%. This outcome strongly confirms the validity and reliability of the developed RVE models. Consequently, for both AS4/8552 with $v_{f}=58\%$ and E-glass/MTM57 with $v_{f}=54\%$, it can be concluded that adopting an RVE with at least 12 fibers is adequate in terms of prediction accuracy for the homogenized elastic properties and damage initiation strengths, while remaining affordable in terms of computational cost.

### 6.3. Models’ Validity in Predicting Failure Modes and Locus

**Figure 10:**
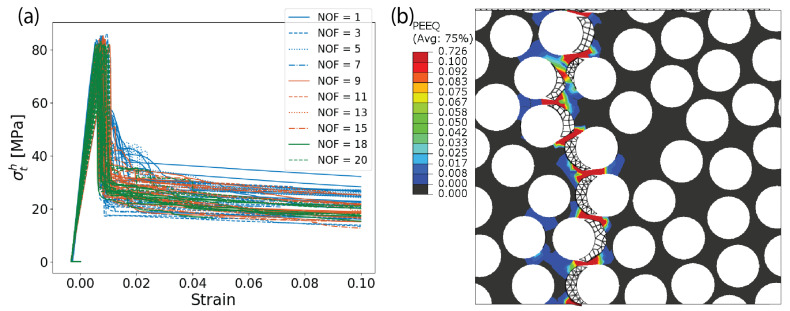

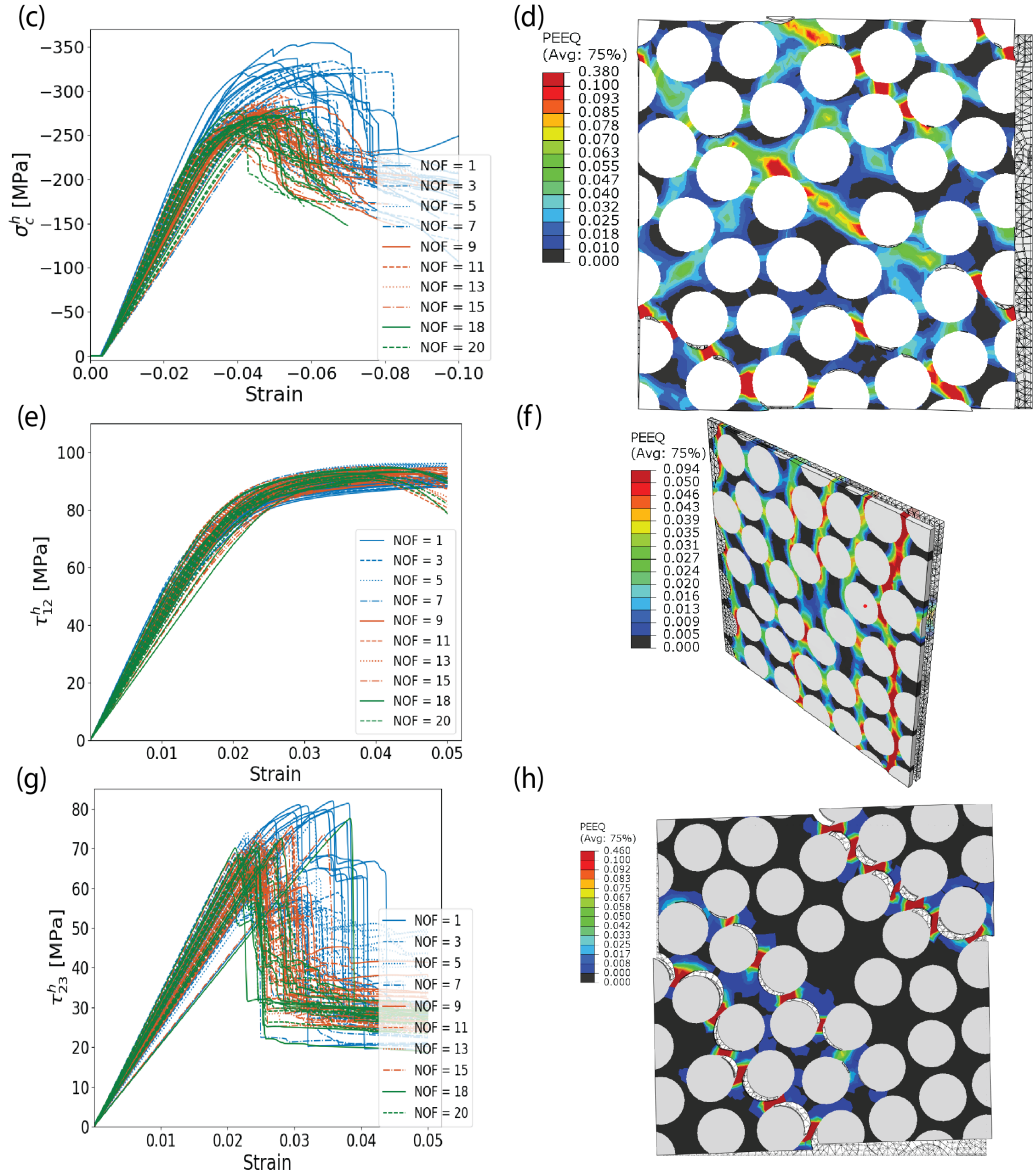
(Color online) Stress–strain curves resulting from the RVE models of AS4/8552, along with the accumulated plastic strain under various loading conditions: (**a**,**b**) transverse tension; (**c**,**d**) transverse compression; (**e**,**f**) longitudinal shear; (**g**,**h**) transverse shear.

**Figure 11:**
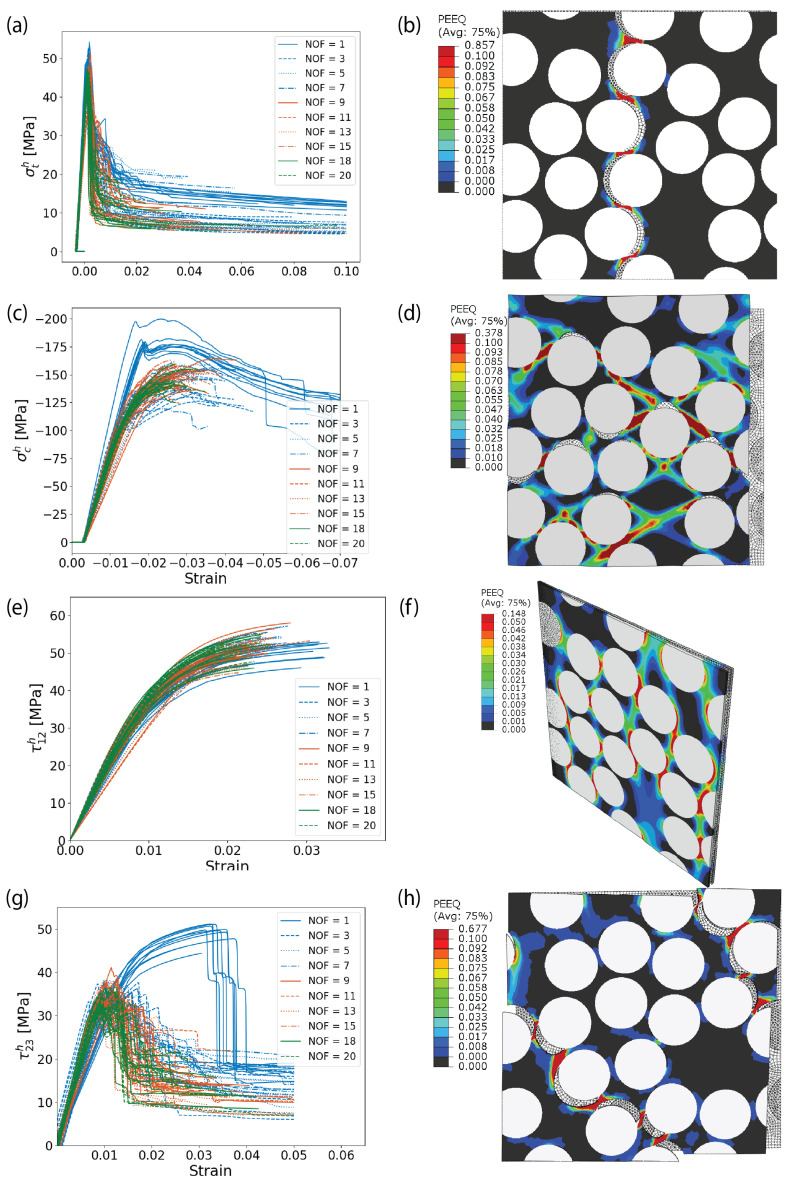
(Color online) Stress–strain curves resulting from the RVE models of E-glass/MTM57, along with the accumulated plastic strain under various loading conditions: (**a**,**b**) transverse tension; (**c**,**d**) transverse compression; (**e**,**f**) longitudinal shear; (**g**,**h**) transverse shear.

For both AS4/8552 and E-glass/MTM57, when subjected to transverse tension, the stress–strain curves depicted in Figure 10a and Figure 11a exhibit an initial nonzero negative strain. This initial strain represents the effects of residual stresses resulting from the heating during the curing process and subsequent cooling. After this point, the behavior is essentially linear elastic until reaching the failure point, where a sudden degradation of stiffness occurs, resembling a brittle failure mode. The predicted failure locus and mechanism are visually represented by the contour of plastic deformation in Figure 10b and Figure 11b. Notably, the initial failure arises in the cohesive elements of the fiber–matrix interface. In other words, the fracture process is primarily driven by the separation of the fiber–matrix interface. Cracks initiate at the fiber poles aligned with the loading direction, particularly in areas of high stress concentrations at the fiber–matrix interface, such as fiber clusters. This illustrates the significant effect of the fiber–matrix interface strength on the transverse tensile strength. After interface failure, the matrix undergoes substantial plastic deformation, progressively accumulating damage until the matrix ligaments fail. The final failure occurs through the development of a crack perpendicular to the loading axis, as seen in Figure 10b and Figure 11b. This behavior is consistent with experimental observations under the microscope.

Under transverse compression, the stress–strain curves depicted in Figure 10c and Figure 11c exhibit a more ductile response compared to those observed in transverse tension. This is logically expected since, in transverse compression, it is not primarily the cohesive elements that fail first or at least they are not the predominant damage mechanism. Instead, significant plastic deformation of the matrix material is observed under compression. As compression continues, debonding occurs at the poles of the fibers. This is due to the fact that the compression strain is applied horizontally, causing the cohesive elements to fail at the upper and lower sides of the fibers. However, the predominant feature is the extensive plastic deformation of the matrix itself. This plastic deformation leads to the formation of plastic bands within the material, which take on the form of diagonal and periodic bands, as shown in Figure 10d and Figure 11d. This behavior aligns well with experimental observations in the literature.

Under longitudinal shear, the debonding of the fiber–matrix interface occurs in shear mode rather than normal mode. This is due to the application of shear stress parallel to the fiber direction, which causes the cohesive elements to fail under shear forces. Additionally, plastic deformation emerges between the fibers within the remaining matrix ligaments, as shown in Figure 10e,f and Figure 11e,f. In contrast, under transverse shear, the scenario is equivalent to applying transverse tension and compression to two perpendicular planes rotated 45 degrees from the initial plane where the transverse shear was applied. This leads to the emergence of a diagonal crack propagating through the fiber–matrix interfaces, as illustrated in Figure 10g,h and Figure 11g,h. These descriptions of the failure locus and mechanisms under transverse and longitudinal shear align well with actual experimental observations. To sum up, the developed RVE models are valid for predicting the failure modes and loci of AS4/8552 and E-glass/MTM57.

## 7. The Effect of *v_f_* on the Size of RVE across Various FRP Types

**Figure 12:**
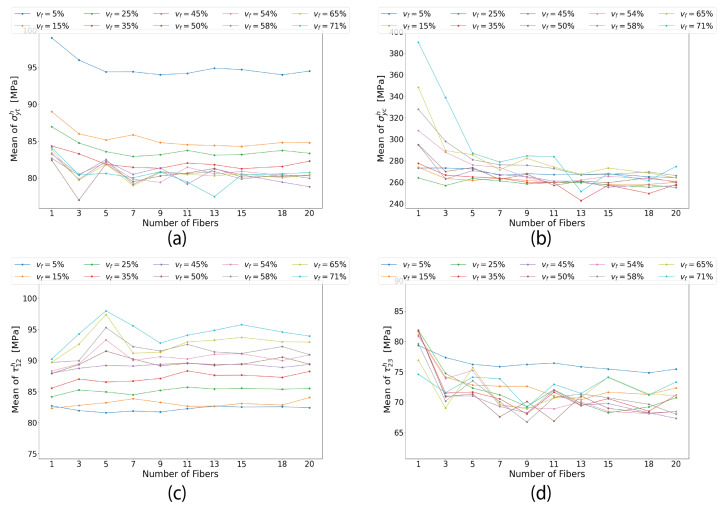

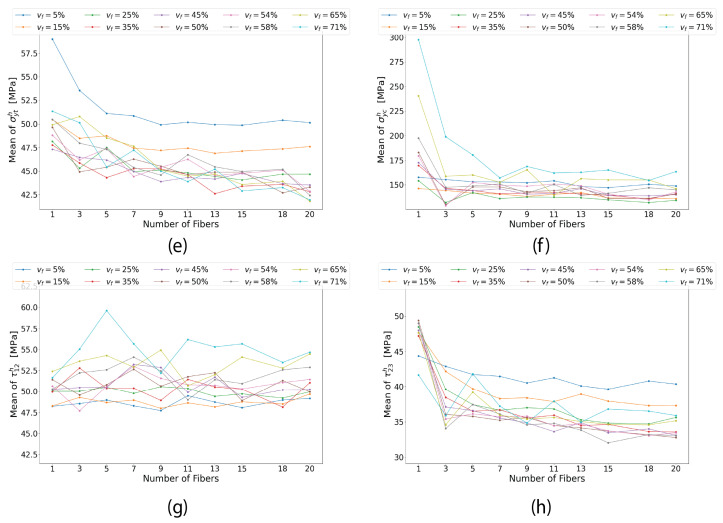
(Color online) Homogenized damage initiation strengths for AS4/8552 plies of different $v_{f}$ and $N_{f}$, (**a**) $\sigma_{yt}^{h}$, (**b**) $\sigma_{yc}^{h}$, (**c**) $\tau_{12}^{h}$, (**d**) $\tau_{23}^{h}$, and for E-glass/MTM57 plies of different $v_{f}$ and $N_{f}$: (**e**) $\sigma_{yt}^{h}$, (**f**) $\sigma_{yc}^{h}$, (**g**) $\tau_{12}^{h}$, (**h**) $\tau_{23}^{h}$.

To investigate the effect of $v_{f}$ on the optimum size of the RVE, the convergence of virtual test results for UD AS4/8552 and E-glass/MTM57 plies with different $v_{f}$ and RVE sizes was studied and analyzed similarly to the method presented in Section 6. Due to space constraints, only the curves of damage initiation strengths are presented in Figure 12, as they fundamentally control the required minimum size of the RVE (i.e., $N_{f}$) that guarantees convergence and low computational cost, as discussed in Section 6. From this figure, it is evident that the convergence of $\sigma_{yt}^{h}$, $\sigma_{yc}^{h}$, $\tau_{12}^{h}$, and $\tau_{23}^{h}$ is achieved at a lower $N_{f}$ value for RVEs with a lower $v_{f}$ compared to those with a higher $v_{f}$. For instance, in the RVEs of AS4/8552 and E-glass/MTM57 with a $v_{f}$ of 5%, employing an $N_{f}$ value of one (unit cell model) or at most three would be sufficient in certain scenarios to predict converged homogenized elastic properties and damage initiation strengths. In contrast, for the RVE of AS4/8552 with a $v_{f}$ as high as 71%, the attainment of converged results necessitates the utilization of an $N_{f}$ value of at least 14.

**Figure 13:**
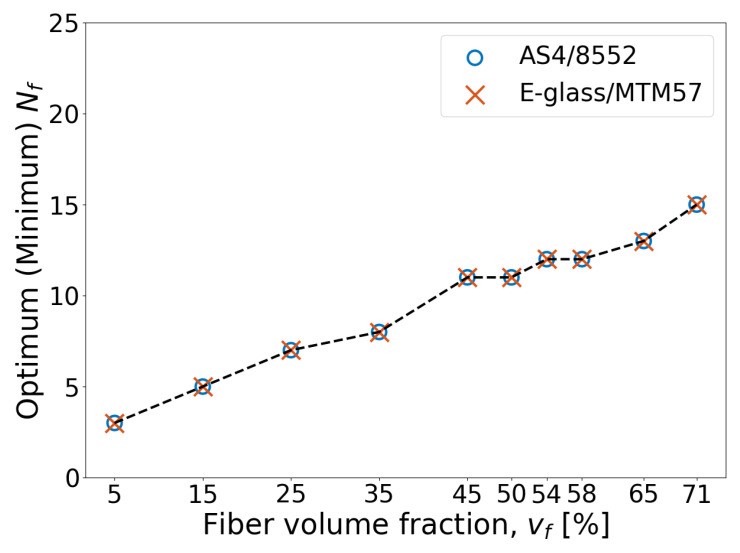
(Color online) The optimum $N_{f}$ in the RVE at each $v_{f}$ for AS4/8552 and E-glass/MTM57.

The optimal size of the RVE, in terms of $N_{f}$, for each $v_{f}$ is graphically represented in Figure 13 for AS4/8552 and E-glass/MTM57. This figure confirms the findings discussed earlier, namely that an increase in $v_{f}$ corresponds to a concurrent increase in the minimum or optimal size of the RVE (i.e., $N_{f}$) required to ensure the convergence of homogenized mechanical properties. This outcome is explicable by the fact that increasing the $v_{f}$ necessitates a larger RVE with a higher $N_{f}$ to encompass the full spectrum of irregularities, such as fiber clustering and resin-rich areas, which significantly influence the predicted stress distribution and mechanical properties. In the same context, when the $v_{f}$ is extremely low, it appears that a unit cell model in some scenarios, or a model with at most two or three fibers, suffices to capture the full spectrum of irregularities. These findings remain valid regardless of the FRP type being modeled using the RVE, whether it be AS4/8552, E-glass/MTM57, or any other type.

## 8. The Effect of *v_f_* on the Reliability and Predictability of Homogenized Elastic Properties and Damage Initiation Strengths across Various FRP Types

To objectively assess the effect of $v_{f}$ on the reliability and predictability of homogenized elastic properties and damage initiation strengths for UD AS4/8552 and E-glass/MTM57, the assessment was implemented in two steps. Firstly, the effect of $v_{f}$ on each of the predicted properties was examined. Subsequently, the effect of $v_{f}$ on the coefficient of variance (CV) of the predicted property was evaluated.

### 8.1. Effect of $v_{f}$ on the Predicted Properties

**Figure 14:**
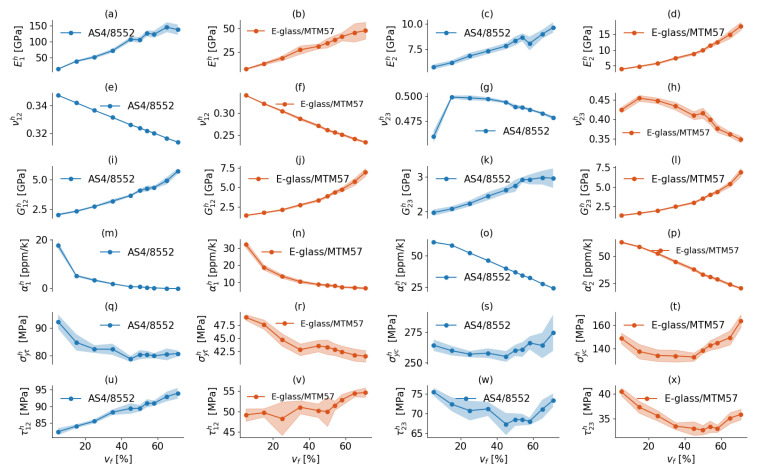
(Color online) The homogenized elastic properties and failure initiation strengths for AS4/8552 and E-glass/MTM57 at each $v_{f}$: (**a**,**b**) $E_{1}^{h}$; (**c**,**d**) $E_{2}^{h}$; (**e**,**f**) $\nu_{12}^{h}$; (**g**,**h**) $\nu_{23}^{h}$; (**i**,**j**) $G_{12}^{h}$; (**k**,**l**) $G_{23}^{h}$; (**m**,**n**) $\alpha_{1}^{h}$; (**o**,**p**) $\alpha_{2}^{h}$; (**q**,**r**) $\sigma_{yt}^{h}$; (**s**,**t**) $\sigma_{yc}^{h}$; (**u**,**v**) $\tau_{12}^{h}$; (**w**,**x**) $\tau_{23}^{h}$.

Taking into account the virtual test results obtained using the RVE models of sizes that guarantee convergence, the mean homogenized elastic properties and damage initiation strengths, accompanied by an error band signifying a 95% confidence interval of the average at each $v_{f}$ level, are depicted in Figure 14 for both AS4/8552 and E-glass/MTM57. It is evident that as $v_{f}$ in the composite increases, the influence of fiber properties becomes more pronounced at the expense of matrix properties. This is clearly observed in Figure 14a–d,i–l for both AS4/8552 and E-glass/MTM57. With an increase in $v_{f}$, the stiffness parameters of the FRP, such as the homogenized Young moduli ($E_{1}^{h}$, $E_{2}^{h}$, and $E_{3}^{h}$) and shear moduli ($G_{12}^{h}$, $G_{13}^{h}$, and $G_{23}^{h}$), also rise. This behavior can be attributed to the higher Young’s and shear moduli of the fiber material in both transverse and longitudinal directions compared to the polymer matrix, as indicated in Table 1 and Table 2. In contrast, Figure 14e–h,m–p illustrates that an increase in $v_{f}$ leads to a decrease in the thermal expansion coefficients ($\alpha_{1}^{h}$, $\alpha_{2}^{h}$, and $\alpha_{3}^{h}$) and Poisson’s ratios ($\nu_{12}^{h}$, $\nu_{13}^{h}$, and $\nu_{23}^{h}$). This decline is driven by the lower thermal expansion coefficients and Poisson’s ratios of the fibers compared to those of the polymer matrix, as indicated in Table 1 and Table 2.

The longitudinal tensile strength in UD FRPs is primarily provided by the fibers; therefore, increasing $v_{f}$ results in an increase in the longitudinal tensile strength. However, for the transverse tensile strength ($\sigma_{yt}^{h}$) of UD FRPs, this property is primarily governed by the matrix strength and the strength of the fiber–matrix interface. Consequently, an increase in $v_{f}$ leads to a reduction in $\sigma_{yt}^{h}$, as seen in Figure 14q,r. This occurs because as $v_{f}$ increases, there are fewer matrix areas available to resist the applied loads, leading to stress concentration areas and a lower load carrying capacity. Notably, the rate of decline in $\sigma_{yt}^{h}$ diminishes as $v_{f}$ increases.

In Figure 14s,t, the behavior of the homogenized transverse compressive strengths ($\sigma_{yc}^{h}$) reveals an interesting trend. Increasing $v_{f}$ up to 45% corresponds to a decrease in $\sigma_{yc}^{h}$. However, beyond this threshold, when $v_{f}$ exceeds 45%, there is a subsequent increase in $\sigma_{yc}^{h}$. This behavior can be explained by the fact that transverse compression in the UD FRP is primarily resisted by the polymer matrix. As $v_{f}$ increases up to 45%, the portion of the polymer matrix available to resist external loads decreases, leading to more stress concentration areas and failure under lower external loads. Conversely, when $v_{f}$ exceeds 45%, the formation of fiber clusters contributes to resisting compressive loads in the transverse direction, resulting in the observed increase in $\sigma_{yc}^{h}$.

In Figure 14u,v, it is apparent that an increase in $v_{f}$ leads to an increase in the longitudinal shear strength ($\tau_{12}^{h}$). This behavior is reasonable because longitudinal shear stresses are predominantly resisted by the shear strength of the fiber-matrix interface and the fibers subjected to tension. As $v_{f}$ increases, the UD FRP’s resistance to longitudinal shear stresses is bolstered, resulting in the observed increase in $\tau_{12}^{h}$.

A UD FRP under transverse shear can be conceptualized as the result of applying transverse tension and compression on two perpendicular planes rotated $45^{\circ }$ from the initial plane where the transverse shear was applied. Consequently, the impact of $v_{f}$ on $\tau_{23}^{h}$ is akin to the combined effect of $v_{f}$ on the transverse tensile and compressive strengths of the UD FRP, as depicted in Figure 14w,x. As $v_{f}$ increases up to 45%, $\tau_{23}^{h}$ decreases. However, when $v_{f}$ exceeds 45%, $\tau_{23}^{h}$ increases. Notably, the rate of increase in $\tau_{23}^{h}$ is lower than that of $\sigma_{yc}^{h}$, owing to the fact that $\tau_{23}^{h}$ is influenced by both the increase in $\sigma_{yc}^{h}$ and the decrease in $\sigma_{yt}^{h}$ as $v_{f}$ rises. To sum up, it is evident that the aforementioned findings and interpretations exhibit consistency and hold true for both of the studied UD FRPs, AS4/8552 and E-glass/MTM57.

### 8.2. Effect of $v_{f}$ on the CV of the Predicted Properties

**Figure 15:**
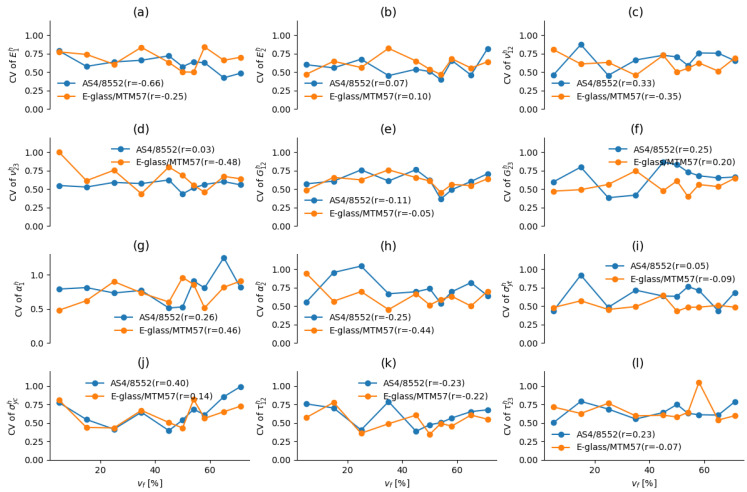
(Color online) The CV of the normalized homogenized elastic properties and failure initiation strengths for AS4/8552 and E-glass/MTM57 at each $v_{f}$: (**a**) $E_{1}^{h}$; (**b**) $E_{2}^{h}$; (**c**) $\nu_{12}^{h}$; (**d**) $\nu_{23}^{h}$; (**e**) $G_{12}^{h}$; (**f**) $G_{23}^{h}$; (**g**) $\alpha_{1}^{h}$; (**h**) $\alpha_{2}^{h}$; (**i**) $\sigma_{yt}^{h}$; (**j**) $\sigma_{yc}^{h}$; (**k**) $\tau_{12}^{h}$; (**l**) $\tau_{23}^{h}$.

The obtained data at each $v_{f}$ were subjected to linear transformation through Min–Max Normalization. Subsequently, the mean and standard deviation of the normalized homogenized elastic properties and damage initiation strengths at each $v_{f}$ were estimated. The coefficient of variation (CV) was then calculated by dividing the standard deviation by the mean. These CVs serve as straightforward indicators to evaluate the reliability and predictability of the effective mechanical properties of UD AS4/8552 and E-glass/MTM57 at various $v_{f}$ levels. In Figure 15, the values of the CV are depicted for the normalized mechanical properties at each $v_{f}$ for both UD AS4/8552 and E-glass/MTM57. Furthermore, the correlation coefficients (*r*) between CVs and $v_{f}$ for each of the normalized mechanical properties are indicated in the legend of each respective subfigure within Figure 15.

For both AS4/8552 and E-glass/MTM57, it is evident from this figure that the values of the CV exhibit fluctuations as $v_{f}$ changes, without displaying a specific trend, whether increasing or decreasing. Consequently, it is challenging to discern any consistent pattern, suggesting that $v_{f}$ does not significantly impact the reliability and predictability of homogenized elastic properties and damage initiation strengths. This conclusion is further supported by the low values of the correlation coefficient *r*, which, in most cases, exhibits |r| values of 0.25 or less. The highest value observed is |r|=0.66 for $E_{1}^{h}$ of AS4/8552, which does not provide substantial insight into the relationship between $v_{f}$ and the reliability and predictability of the homogenized mechanical properties and damage initiation strengths.

Therefore, it can be deduced that while $v_{f}$ indeed has a direct influence on the values of homogenized elastic properties and damage initiation strengths, it does not appear to have a distinct effect on the reliability and predictability of these properties. The latter aspect is more affected by the inherent randomness and uncertainties in the properties of FRP constituents (fibers and matrices) and from defects that arise during the manufacturing process of FRP composites, such as voids in the matrix, uneven fiber spatial distribution, fiber misalignment, and fiber waviness. These conclusions hold true for both AS4/8552 and E-glass/MTM57.

## 9. Conclusions

This research employed a micromechanical virtual testing approach using representative volume element (RVE) models to primarily investigate the influence of $v_{f}$ on the predictability and reliability of the homogenized elastic properties and damage initiation strengths for UD AS4/8552 and E-glass/MTM57 plies. Additionally, the study explored the effect of $v_{f}$ on the optimum sizes of the RVE in terms of fiber number ($N_{f}$). For this purpose, micromechanical RVE models were developed and validated against actual experimental data. A modified version of an algorithm based on a constrained optimization formulation was developed to generate random microstructures for the RVE models. The models considered the inherent uncertainties in constituent properties, random fiber arrangement, and fiber diameter, making the investigation more realistic and resemble actual testing. The required data to model each of these uncertainties were obtained from the literature and fitted to a probability density function (PDF). The virtual tests were systematically conducted using full factorial design of experiments (DOE) coupled with Monte Carlo simulation. The findings can be categorized and summarized in the following points:

- The modified algorithm based on a constrained optimization formulation exhibited exceptional performance regarding convergence speed and jamming limit, which reached 77%. It took less than 5 minutes on average to generate a feasible microstructure of size 50×50 with a $v_{f}$ of 65%, which is lower than the time required by the original algorithm (18 minutes) to generate a microstructure with the same size and $v_{f}$. This is highly beneficial for uncertainty analysis using virtual testing, particularly when many microstructures need to be generated.
- Increasing $v_{f}$ leads to a concurrent increase in the minimum or optimal size of the RVE (i.e., $N_{f}$) required to ensure the convergence of homogenized mechanical properties. This outcome is explicable by the fact that an increase in $v_{f}$ necessitates a larger RVE with a higher $N_{f}$ to encompass the full spectrum of irregularities, such as fiber clustering and resin-rich areas, which have a pronounced influence on the predicted stress distribution and mechanical properties. Conversely, when $v_{f}$ is extremely low, it seems that a unit cell model in some scenarios or a model of two or three fibers suffices to include the full spectrum of irregularities.
- Increasing $v_{f}$ in fiber-reinforced polymers (FRPs) accentuates the influence of fiber properties on the composite’s properties. In cases like UD AS4/8552 and E-glass/MTM57, the fibers’ stiffnesses ($E^{f},G^{f}$) surpassed that of the matrix, leading to an overall increase in the stiffness of the composite. Conversely, the Poisson’s ratios and thermal expansion coefficients of the fibers were lower than those of the matrix, causing a decrease in these properties for the FRPs with increasing $v_{f}$.
- Regarding damage initiation strengths, increasing $v_{f}$ in UD AS4/8552 and E-glass/MTM57 resulted in higher longitudinal tensile and shear strengths, but lower transverse tensile strength due to reduced matrix areas available to resist loads, leading to the creation of stress concentration areas and, consequently, a lower load carrying capacity. Transverse compressive and shear strengths decreased initially with the increase in $v_{f}$ up to a specific threshold (45%), but increasing $v_{f}$ beyond this threshold corresponded to an increase in these strengths. This could be attributed to the formation of fiber clusters contributing to resisting the compressive load in the transverse direction.
- While $v_{f}$ indeed had a direct influence on the values of homogenized elastic properties and damage initiation strengths, it did not appear to have a distinct or significant effect on the reliability and predictability of these properties. This conclusion was substantiated by the low values of the correlation coefficient (*r*) and the observed fluctuations in the value of the CV of the normalized properties as $v_{f}$ changed.

## Figures and Tables

**Table 1:** The mechanical properties of AS4 and E-glass fibers [19,56,59].

Fiber	$E_{1}^{f}$ [GPa]	$E_{2}^{f}$ [GPa]	$G_{12}^{f}$ [GPa]	$G_{23}^{f}$ [GPa]	$\nu_{12}^{f}$	$\alpha_{1}^{f}$ [ppm/K]	$\alpha_{2}^{f}$ [ppm/K]	$X_{t}^{f}$ [MPa]	$X_{c}^{f}$ [MPa]
AS4	231	12.9	11.3	4.45	0.3	−0.7	12	4000	3500
E-Glass	74	74	30.8	30.8	0.2	4.9	4.9	2150	1450

**Table 2:** The mechanical properties of 8552 and MTM57 epoxies [13,14,18,20,56].

Epoxy	$E^{m}$ [GPa]	$\nu^{m}$	$\alpha^{m}$ [ppm/K]	$\sigma_{t}^{m}$ [MPa]	$\sigma_{\mathrm{yc}}^{m}$ [MPa]	$\sigma_{\mathrm{uc}}^{m}$ [MPa]	$G_{I}^{m}$ [J/m^2^]	$\psi^{m}$ [°]
8552	5.1 ± 0.3	0.35	52	120	176 ± 3	180	90	29 ± 1
MTM57	3.5 ± 0.2	0.35	58	≥75	105 ± 5	-	-	-

**Table 3:** The fiber–matrix interface properties of AS4/epoxy and E-glass/epoxy [13,14,20,56].

Interface	$N^{c}$ [MPa]	$S^{c}$ [MPa]	$G_{n}^{c}$ [J/m^2^]	$G_{s}^{c}$ [J/m^2^]	$k_{n}^{c}$ [GPa/μm]	$k_{s}^{c}$ [GPa/μm]
AS4/epoxy	57	85	7	80	100	100
E-glass/epoxy	50	75	2	10	100	100

**Table 4:** Factors and levels of the full factorial DOE for virtual testing.

Factors	Levels
$v_{f}$ [%]	10 Levels (5, 15, 25, 35, 45, 50, 54, 58, 65, 71)
RVE size, $N_{f}$	10 Levels (1, 3, 5, 7, 9, 11, 13, 15, 18, 20)

**Table 5:** The effective properties of macroscopic coupons extracted from UD AS4/8552 laminate and UD E-glass/MTM57 laminate [20,59,74,75,76,77,78,79].

Composite Type	$E_{1}$ [GPa]	$E_{2}=E_{3}$ [GPa]	$\nu_{12}=\nu_{13}$	$\nu_{23}$	$G_{12}=G_{13}$ [GPa]	$G_{23}$ [GPa]	$\alpha_{1}$ [ppm/K]	$\alpha_{2}=\alpha_{3}$ [ppm/K]	$Y_{t}$ [MPa]	$Y_{c}$ [MPa]
AS4/8552	141	9.2	0.302	0.49	5.2	3.19	0~−0.8	31.2	81	260
E-glass/MTM57	45.6	11	0.27	0.4	3.88	3.93	8.6	35	45	145

## Data Availability

The raw data supporting the conclusions of this article will be made available by the authors on request.
